# Release Mechanism, Secondary Pollutants and Denitrification Performance Comparison of Six Kinds of Agricultural Wastes as Solid Carbon Sources for Nitrate Removal

**DOI:** 10.3390/ijerph18031232

**Published:** 2021-01-29

**Authors:** Yu Ling, Guokai Yan, Haiyan Wang, Weiyang Dong, Huan Wang, Yang Chang, Ming Chang, Congyu Li

**Affiliations:** 1 Research Center of Environmental Pollution Control Technology, Chinese Research Academy of Environmental Science, Beijing 100012, China; lingyu18@mails.ucas.ac.cn (Y.L.); yangk@craes.org.cn (G.Y.); docreat@163.com (W.D.); wanghuan989193@126.com (H.W.); cy1100@126.com (Y.C.); licongyu1996@163.com (C.L.); 2 Basin Research Center for Water Pollution Control, Chinese Research Academy of Environmental Science, Beijing 100012, China

**Keywords:** agricultural wastes, solid carbon source, release mechanism, secondary pollutants, denitrification

## Abstract

Agricultural wastes used as denitrification carbon sources have some drawbacks such as excessive organic carbon release and unclear release characteristics of nitrogen, phosphorus, and chromatic substances, which can cause adverse effects on the effluent quality during the denitrification process. The composition and surface characteristics, carbon release mechanisms, and secondary pollutant release properties of six kinds of agricultural wastes, i.e., rice straw (RS), wheat straw (WS), corn stalk (CS), corncob (CC), soybean stalk (SS), and soybean hull (SH) were studied and analyzed in this research. The denitrification performance of these agricultural wastes was also investigated extensively by batch experiments. The results showed that the carbon release basically followed the second-order reaction kinetic equation and Ritger–Peppas equation in the 120 h reaction, and it was mainly controlled by the diffusion process. The kinetic equation fitting results and bioavailability test suggested that the potential risk of excessive effluent COD of CC was the lowest due to the appropriate amount and degradability of its released carbon. The NH_4_^+^-N, TN, and TP in the leachate of RS were higher than those of the other five agriculture wastes, and the chroma in the leachate of WS and CS was heavier than that of the others. CC released the lowest pollutants, which resulted in slight fluctuations of effluent quality in the start-up period (1–11 d), and it had the best nitrogen removal capacity in the denitrification experiment. The average NO_3_^−^-N removal of CC was 5.12 mg for each batch in the stable period (11–27 d), which was higher than that of others, and less NO_2_^−^-N, NH_4_^+^-N, and COD were accumulated in the CC effluent during the whole denitrification process.

## 1. Introduction

Nitrogen pollution has become a severe issue due to rapid socio-economic development [1]. Large amounts of nitrogen compounds are discharged into the water due to excessive fertilizer application and domestic and industrial discharge [2]. Excessive nitrogen can cause serious environmental problems, such as the as eutrophication of rivers and lakes and the deterioration of water sources, and then influence human health [3]. Therefore, nitrogen removal from water and wastewater is important for environmental and human health protection.

Among all the nitrogen removal methods and technologies, biological denitrification is generally applied due to its high efficiency and low cost. Heterotrophic denitrification, which utilizes organic carbon sources as electron donors, has higher denitrification rates compared with autotrophic denitrification [4]. However, the deficiency of available carbon sources is an intractable problem for heterotrophic denitrification in the treatment of wastewater with low carbon to nitrogen (C/N) ratio such as wastewater treatment plants (WWTP) effluent, rural sewage, farmland recession, landfill leachate, and industrial effluents [5,6,7,8], etc. Conventionally, liquid carbon sources as methanol, ethanol and acetic acid are applied in the treatment of low C/N ratio wastewater to ensure sufficient electron donors for the denitrification process [9]. The dosage of liquid carbon source is difficult to control, which leads to the deterioration of the effluent quality, and sophisticated control and continuous monitoring processes are always required, thus increases the operational costs [10].

In order to solve the problems mentioned above, a novel process named solid-phase denitrification (SPD) was developed, which utilizes natural lignocellulosic materials and synthetic biodegradable polymers as carbon sources and biofilm carriers [11,12,13,14]. The biodegradable cellulose and hemicellulose substances of the natural lignocellulosic materials can be hydrolyzed into soluble and small molecular organic matter by the extracellular enzymes excreted by the microbes attached on the biofilm, and then supplied as electron donors for the denitrification process [15,16]. At present, most research on lignocellulosic materials as carbon sources are focused on the selection of high-efficiency materials, pretreatment methods to enhance nitrate removal capacity, and microbial community succession in the nitrogen removal process [17,18,19,20]. Agricultural wastes, which are mainly composed of lignocellulosic materials, are also widely studied because of their cheapness and easy availability [16]. However, the contrasting research about the nitrogen removal efficiency of different agricultural wastes as denitrification carbon sources is insufficient. Meanwhile, issues such as the excessive release of organic matter and secondary pollutant discharge in the initial stage, high chroma of the effluents, and the long start-up period have not been investigated effectively. In practice, great fluctuation of the effluent quality might be caused by the addition and replacement of agricultural wastes; moreover, the release characteristics of the carbon source and secondary pollutants are unclear. These problems impede the practical application of agricultural wastes as denitrification carbon sources.

This study aims to reveal the release characteristics of carbon sources and secondary pollutants from the agricultural wastes, and to investigate the influence of agricultural wastes and their composition and surface properties on nitrate removal from wastewater. Six kinds of agricultural wastes were tested and compared by batch experiments in order to find an appropriate and efficient one for the treatment of wastewater with a low C/N ratio. The results can provide theoretical support for the selection of highly efficient carbon sources, the prevention of secondary pollution in practical application and the potential risk assessment of agricultural wastes as denitrification carbon sources.

## 2. Materials and Methods 

### 2.1. Materials

The raw materials were obtained from a peasant household in the countryside of Linyi, Shandong, China. The collected rice straw (RS), wheat straw (WS), corn stalk (CS), corncob (CC), soybean stalk (SS), and soybean hull (SH) were dried naturally and cut into similar pieces about 1 cm.

### 2.2. Release Experiments

Agricultural wastes of 3 grams and 250 mL ultra-pure water were added into 250 mL Erlenmeyer flasks, which were incubated statically for 5 days. Two sets of parallel samples were taken after 1, 3, 6, 12, 24, 36, 48, 72, 96, and 120 h, and the chemical oxygen demand (COD), total organic carbon (TOC), nitrate (NO_3_^−^-N), nitrite (NO_2_^−^-N), ammonia-N (NH_4_^+^-N), total nitrogen (TN), total phosphorus (TP), and chroma were measured to study the release characteristics of carbon source and secondary pollutants. The soaking solution was replaced every time after sampling.

The second-order reaction kinetic equation and Ritger–Peppas release equation were fitted to the carbon release process of the experimental agricultural wastes. The second-order reaction kinetic can be expressed by:

(1) $$dc/dt=kc^{2},$$

Rearrangement of Equation (1) yields

(2) $$1/c-1/c_{m}=k\;/t,$$

In Equation (2):

(3) $$1/k=\;c_{m}/t_{1/2\;},$$

where *c* is the accumulated COD in time *t*, mg/(g·L); *c*_m_ is the ultimate accumulated COD, mg/(g·L); *k* is the constant of carbon release; *t* is time, h; *t*_1/2_ is the time needed for the carbon release to half of its maximum concentration.

The Ritger–Peppas equation is:

(4) $$M_{t}/M_{\infty }=kt^{n},$$

where *M_t_* is the accumulated COD concentration in time *t*, mg/(g·L); *M_∞_* is the ultimate accumulated COD, mg/(g·L); k is the constant of carbon release; *n* is the carbon release index, which could represent the mechanism of carbon release. The diffusion process is dominant in carbon release when *n* is less than 0.45, the diffusion and disintegration processes are dominant when *n* ranges from 0.45 to 0.89, and the disintegration process is dominant when *n* is higher than 0.89 [21].

### 2.3. Nitrogen Removal Experiments

Raw materials of 3 grams, 100 mL activated sludge (obtained from the anoxic tank of Beijing Xiaojiahe wastewater treatment plant) and 300 mL synthetic water were put into 500 mL flasks and then inoculated. Meanwhile, the flask without agricultural waste addition was set as control (CK). The initial condition of the systems was 1000 mg/L MLSS, 50 mg/L nitrate, and 5 mg/L TP. Then these experiment devices were cultivated in an incubator at 25 ℃ and 100 rpm. The COD, NO_3_^−^-N, NO_2_^−^-N, NH_4_^+^-N, and TN of the supernatant of each flask were measured and analyzed on the first day to investigate the bioavailability of the agricultural wastes’ leachates. Then the samples were taken every two days for the measurement and analysis of the water quality, thus revealing the enhancement of the denitrification process by agricultural wastes as solid carbon sources. We replaced 300 mL supernatant in each flask with influent with 50 mg/L nitrate and 5 mg/L TP after each sampling. The dissolved oxygen was reduced to 0.5 mg/L by N_2_ flushing. The experiments were conducted in two parallel groups.

### 2.4. Characterization and Analytical Methods

#### 2.4.1. Composition Analysis

Clean agricultural wastes were oven-dried at 40 °C to constant weight, milled and screened through a 60-mesh sieve, and then analyzed by Van Soest’s method [22] to quantify cellulose, hemicellulose and lignin content. Total carbon (TC) and TN of agricultural wastes were determined by an elemental analyzer (Thermo Flash 2000 CHNS/O, Thermo Fisher Scientific, Waltham, Massachusetts, USA), and TP of agricultural wastes was analyzed by an Inductively Coupled Plasma Optical Emission Spectrograph (ICP-OES optima 8000, Perkin Elmer, Waltham, MA, USA).

#### 2.4.2. SEM and Water Quality Determination

The surface property of agricultural wastes was observed by a scanning electron microscope (SEM, S570, Hitachi Co., Tokyo, Japan), and the specific steps followed Zhao’s report [23].

COD, NH_4_^+^-N, TP, and MLSS were analyzed according to the standard methods [24], and TP was measured by the ammonium molybdate spectrophotometric method. NO_3_^−^-N and NO_2_^−^-N were analyzed using an ion chromatography (DIONEX ICS-1000, Dionex Inc., Sunnyvale, CA, USA), TOC and TN were measured with a total organic carbon analyzer (TOC-VCPH, Shimadzu, Kyoto, Japan) after 0.45 μm syringe tip-filter filtration (SCAA-201), and chroma was analyzed according to the national standard method (GB 11903-89).

## 3. Results and Discussion

### 3.1. Characteristics of the Studied Materials

#### 3.1.1. Composition of the Investigated Agricultural Wastes

C, N, and P in agricultural wastes demonstrate not only the release capacity of carbon sources but also the potential risk of secondary pollution. As shown in Table 1, the C contents in the raw materials ranged from 43.88% to 69.84%, which indicated that these agricultural wastes were feasible as denitrification carbon sources. WS had the highest C content, followed by RS, CS, SH, CC, and SS. The content of N (14.38%) and P (0.22%) in RS was the highest, which may have released more N and P pollutants and caused adverse effects on the effluent quality. By contrast, CC had the lowest potential risk of secondary pollution.

**Figure 1:**
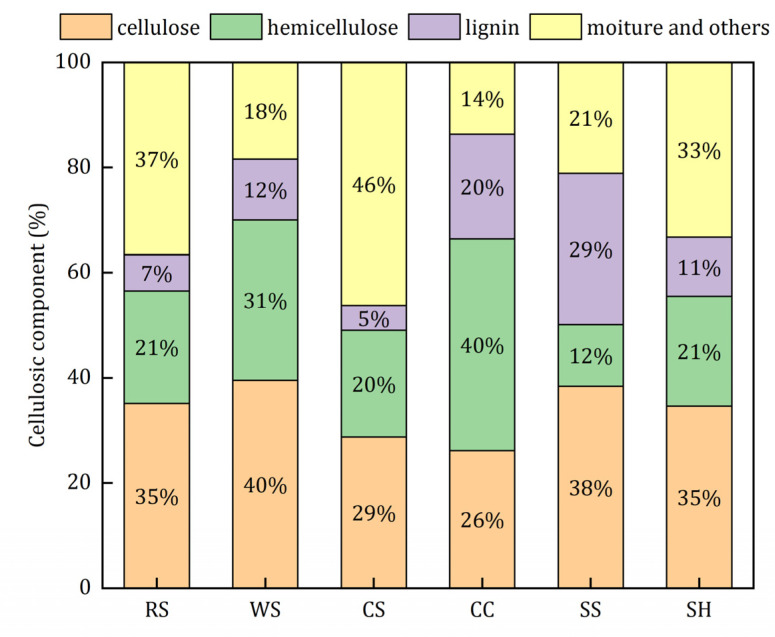
Cellulosic components of the investigated agricultural wastes.

The main constituents of agricultural wastes are biodegradable cellulose, hemicellulose, and refractory lignin [18]. The extracellular enzymes excreted by the microorganisms can hydrolyze cellulose and hemicellulose into soluble and small molecule substrates, which can be utilized by microbes during the denitrification process [25]. The supply capacity and service life of carbon sources are related to the content of the biodegradable constituents. It can be seen from Figure 1 that WS and CC had more cellulose and hemicellulose than the other materials. Theoretically, WS and CC could provide more electron donors continuously. The corn stalk had the lowest effective carbon source content.

#### 3.1.2. Surface Property of the Investigated Agricultural Wastes

**Figure 2:**
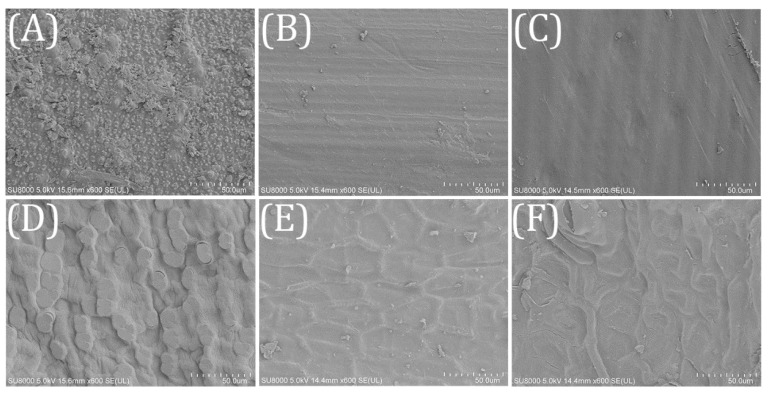
SEM for the investigated agricultural wastes (×500) (**A**) RS; (**B**) WS; (**C**) CS; (**D**) CC; (**E**) SS; (F) SH.

The surface structure of the biofilm carriers has a significant impact on the attachment and growth of functional bacteria communities [26]. The surface property of agricultural wastes was analyzed by SEM. As shown in Figure 2, RS surface was rough and covered with small granular protrusions, which might be beneficial to the microbial adhesion. Both WS and CS had smooth surface, and a densely lignocellulosic structure arranged in stripes was observed. CC also had a rough surface. Irregular bulges could provide a larger attachment area for microbes. The surfaces of SS and SH were covered with a lignocellulosic framework, which might restrict the hydrolysis and utilization of cellulose and hemicellulose. Compared with SS, the lignocellulosic framework of SH was more complex and incoordinate.

### 3.2. Release Characteristics of Carbon Sources

#### 3.2.1. Kinetic Characteristics of the Carbon Sources

**Figure 3:**
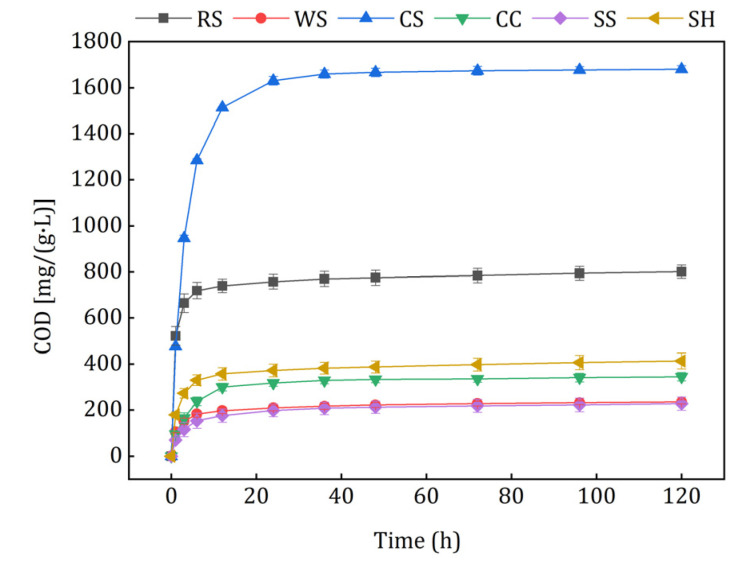
Carbon release curves of the investigated agricultural wastes.

The carbon release curves of different agricultural wastes are shown in Figure 3. The cumulative COD released by CS, RS, SH, CC, WS, and SS were 1680.84, 801.31, 413.45, 344.83, 235.49, and 227.69 mg/(g·L). The raw materials had similar carbon release properties, but different amounts of the carbon sources were released. In the initial 6 h, the cumulative amount of the carbon sources increased quickly, and then the release velocities stabilized at very low levels in 6–120 h. Some potential explanations for this phenomenon are as follows: besides cellulose, hemicellulose and lignin, the natural lignocellulosic materials also contained large amounts of soluble and small molecular organic matter [27,28], which were released rapidly in the initial stage because of the concentration difference. When the inner small molecular substances were exhausted, insoluble cellulose and hemicellulose were hydrolyzed slowly to soluble organics by the microbes, which adhered to the raw materials with low biomass.

The kinetic equation and its parameters are listed in Table 2. The carbon release processes were highly in accord with the second-order reaction and the Ritger–Peppas equation. Small *t*_1/2_ values indicated that the agricultural wastes could release large amounts of carbon sources in a short time. Higher *c*_m_ values of CS and RS meant that they contained more soluble and small molecular organics than the others, and they caused carbon source waste and excessive effluent COD in the initial denitrification process more easily. On the contrary, WS and SS with lower *c*_m_ values might release insufficient carbon source for the utilization of denitrifiers, which could have caused problems such as slow growth of biofilms, bad nitrogen removal performance, and a long start-up period. The *c*_m_ values of CC and SH were moderate while the *t*_1/2_ value of CC was higher, which indicated that the amount and velocity of CC carbon release were appropriate and may have alleviated the problems mentioned above. It can be seen from the fitting results of the Ritger–Peppas calculation that all of the release indexes were less than 0.45, which indicated that the lignocellulosic framework of the selected carbon sources was stable. The agricultural wastes released small molecular organic matters and then transferred these to the solution by diffusion, which was in accord with the study about peanut shells, corncob, and obsolescent rice [29].

#### 3.2.2. Biodegradability of Leachates of the Investigated Agricultural Wastes

**Figure 4:**
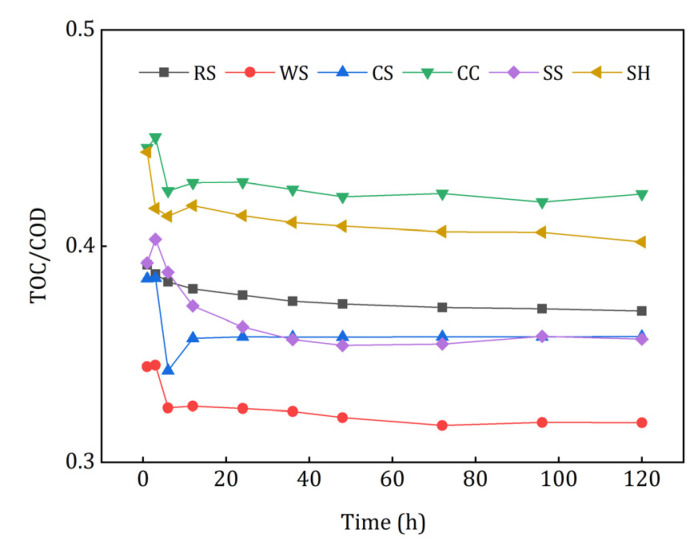
TOC/COD of the investigated agricultural wastes.

COD refers to the amount of oxidant consumed by reducing substances that are easily oxidized by strong oxidants [29], and TOC reflects the amount of the released organic carbon more accurately. As shown in Figure 4, there is an obvious liner relationship between TOC and COD. The TOC/COD of CC leachate was the highest, which demonstrated that the relative organic carbon released from CC was the largest. Conversely, WS had the lowest TOC/COD value, which may have caused inefficient denitrification and excessive COD in the effluent.

**Figure 5:**
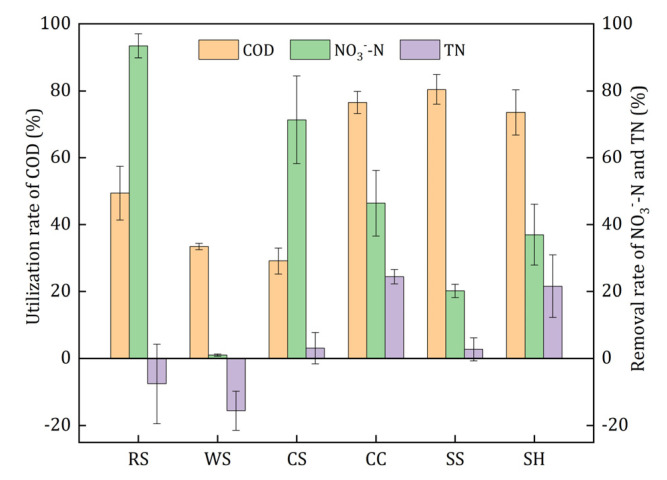
Denitrification performance of leachates of the investigated agricultural wastes.

In order to investigate whether the organic carbon released from agricultural wastes in the initial stage could be utilized by the denitrifiers effectively, a one-day denitrification experiment using the leachate as carbon source was conducted, and the results are illustrated in Figure 5. The utilization rate of COD represented the biodegradability of the leachate, and the removal rate of NO_3_^−^-N and TN indicated the capacity of leachate as denitrification electron donors. The utilization rates of COD ranked in descending order were as follows: 80.41% ± 4.41% (SS), 76.50% ± 3.35% (CC), 73.57% ± 6.79% (SH), 49.40% ± 8.01% (RS), 33.45% ± 0.94% (WS), and 29.14% ± 3.86%(CS). The leachate of CC and SH obtained relatively higher TN removal rates, which means that their released carbon could be used by denitrifying bacteria more easily than that of the other wastes. Although RS and CS leachate had the highest nitrate removal rate, their TN removal was quite low and even negative. On the one hand, RS and CS released large amount of nitrogenous substances; on the other hand, the following denitrification process was incomplete, thus it caused the accumulation of NO_2_^−^-N. Therefore, the leachate of RS and CS was poorly available for the denitrifiers. Slight nitrate removal (0.94% ± 0.29%) was obtained by the leachate of WS, which indicated that it was not suitable as a carbon source. In conclusion, the leachate of CC and SH had better biodegradability and denitrification performance and lower risk of secondary pollution in the initial denitrification process.

### 3.3. Release Characteristics of Secondary Pollutants

**Figure 6:**
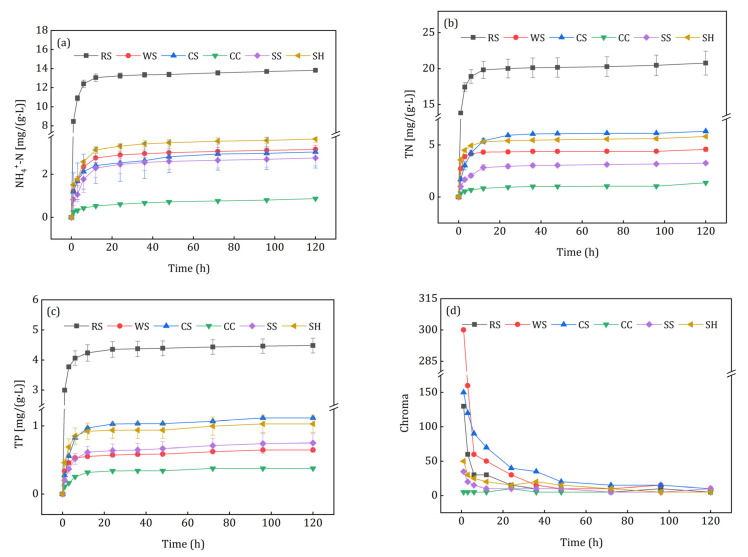
Release curves of secondary pollutants from the investigated agricultural wastes: (**a**) NH_4_^+^-N; (**b**) TN; (**c**) TP; (**d**) chroma.

Figure 6 illustrates the release characteristics of secondary pollutants as N, P, and chroma from agricultural wastes. N and P released rapidly in the initial 6 h, and then the release rate dropped to very low levels in 6–120 h. As shown in Figure 6a, the largest accumulative NH_4_^+^-N [(13.82 ± 0.09) mg/(g·d)] was achieved by RS leachate, and the lowest [(0.87 ± 0.02) mg/(g·d)] occurred in CC leachate. Only RS, WS, and SH released small amounts of nitrates at 1 h, i.e., 0.67 ± 0.31, 0.37 ± 0.26, and 0.09 ± 0.04 mg/(g·d) (Supplementary Figure S1). It can be seen from Figure 6b that the TN release of RS [(20.77 ± 1.67) mg/(g·d)] was significantly higher than that of the other five materials. In practical applications, the superfluous TN released from agricultural wastes may lead to incomplete denitrification and excessive nitrogen discharge [1]. The TP release from different carbon sources is shown in Figure 6c, the maximum released TP was observed in RS leachate while the minimum occurred in CC leachate, which had the same characteristic as NH_4_^+^-N and TN. Figure 6d shows that the leachate of all agricultural wastes had obvious chroma except for CC, and WS and CS leachate had deeper colors than the others in the first 2 days. All of the leachate colors were light yellow, which may result from the chromophore groups of organic matters released from the investigated agricultural wastes [30].

The release of N, P and chroma varied with different crops and their cultivation conditions, which could have affected the denitrification process and increased the difficulty of subsequent treatment; moreover, excessive TN and TP easily occurred in the initial effluent. Aslan et al. [31] and Cao et al. [32] observed heavy effluent chroma during the study of agricultural wastes as additional carbon sources for denitrification. Therefore, secondary pollutants should be considered when using agricultural wastes as denitrification carbon sources and biofilm carriers. Compared with other agricultural wastes, CC was more suitable for denitrification as an additional carbon source.

### 3.4. Denitrification Performance

**Figure 7:**
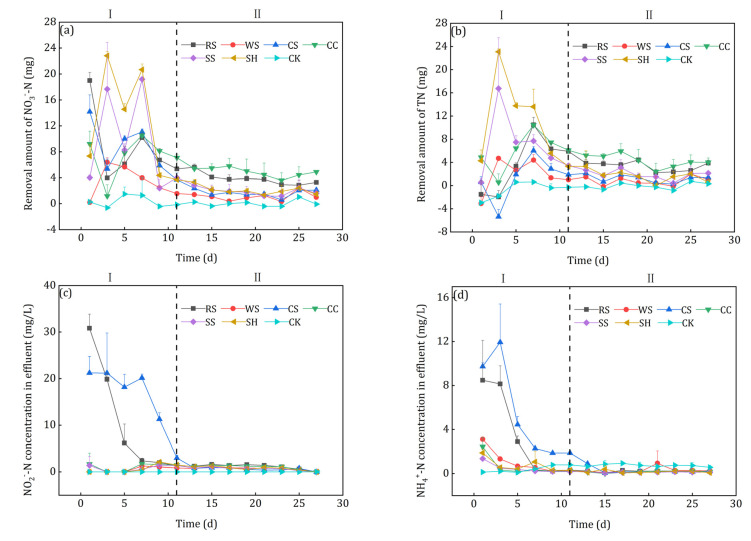

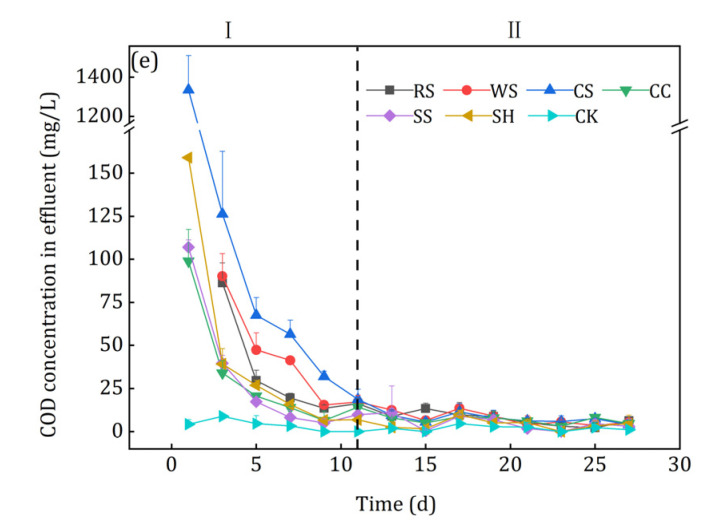
Nitrate removal performance of the investigated agricultural wastes: (**a**) Nitrate removal; (**b**) TN removal; (**c**) Nitrite accumulation; (**d**) NH^4^_+_-N accumulation; (**e**) COD accumulation.

The nitrate removal performance of the 6 kinds of agricultural wastes is shown in Figure 7. Compared with CK, the addition of agricultural wastes had obvious enhancement in the nitrate removal process. It can be seen from Figure 7a that the denitrification process could be divided into two phases, i.e., the start-up phase (phase I, 1–11 d) and the stable phase (phase II 11–27 d). In phase I, the nitrate removal by denitrification fluctuated due to the acclimation of microorganisms, which was similar to the research results of Hang et al. [33]. Initially, the agricultural wastes released abundant soluble and small molecular organics for the denitrification process, but different nitrate removal performance was achieved because of the different amounts and biodegradability of the carbon sources. As discussed in section 3.2., WS had almost no nitrate removal ability on the first day. After the first sampling, the decrease in small molecular organic carbon caused by influent replacement led to the sharp decline of the nitrate removal capacity of RS, CS, and CC. Kumar et al. [34] reported that physical crushing could enhance the bioavailability of plants. The enhanced nitrate removal of WS, SS and SH might be result from the decomposition of the easily degradable small scraps attached to the surface of the agricultural wastes (Figure 2). Then the denitrification came into the stable phase with the gradual maturation of biofilms and the stable utilization of carbon sources. In phase II, the average removal amount of nitrate for each batch were 5.12 mg (CC), 3.94 mg (RS), 2.23 mg (SH), 2.11 mg (SS), 1.86 mg (CS), and 1.14 mg (WS). CC had the best nitrate removal ability because of its rough surface and high proportion (66%) of degradable hemicellulose and cellulose. Although WS had sufficient available carbon sources, its surface was too smooth to attach the hydrolysis and denitrifying bacteria (Section 3.1), which led to the unsatisfactory nitrate removal performance. 

The shape of the TN removal curves was similar to that of the nitrate removal curves after agricultural waste addition (Figure 7b), but a slight difference was observed in the initial stage. The TN removal amounts of RS and CS, whose nitrate removal efficiency was quite high, were only −1.51 mg and 0.61 mg on the first day and then decreased in the following two days. Nitrite severely accumulated in the flasks to which RS and CS was added (Figure 7c), which indicated that incomplete denitrification occurred in phase I. Because of the NH_4_^+^-N release (Figure 6a), it accumulated in the effluents of all 6 kinds of agricultural wastes ((Figure 7d), especially for RS and CS. Rahman et al. [35] reported that dissimilatory nitrate reduction to ammonium (DNRA) tended to occur when the electron donor (COD) was much higher than its accepter (NO_3_^−^-N). Considering the high effluent COD of CS leachate (Figure 7e) and NH_4_^+^-N content in the raw agricultural wastes, the DNRA might be the reason of the highest NH_4_^+^-N in the effluent of CS leachate. In phase II, the effluent NO_2_^−^-N, NH_4_^+^-N and COD were quite low, i.e., 0.78–1.24, 0.12–0.44 and 4.31–9.80 mg/L, respectively. 

Compared with the other agricultural wastes, CC had the advantages of more excellent and stable nitrate and TN removal capacity, less nitrite accumulation, and less effluent NH_4_^+^-N and COD, which was much more appropriate for the solid carbon source for the denitrification process.

## 4. Conclusions

(1) The C content of RS, WS, CS, CC, SS, and SH ranged from 43.88% to 69.84%, and WS had the highest content. CC had the lowest potential risk of secondary pollution when used as a carbon source for denitrification. The degradable constituents of WS and CC were 71% and 66%, which were higher than those of the other agricultural wastes. RS and CC had rougher surfaces than the other wastes, which might be beneficial for microbial adhesion.

(2) The carbon release process of the six kinds of agricultural wastes followed the second-order reaction kinetic equation, and CC had appropriate *c*_m_ and *t*_1/2_ which were beneficial for shortening the start-up period and avoiding excessive COD in the effluent. The carbon release process also satisfied the Ritger–Peppas equation, and the diffusion mechanism dominated the release process. The biodegradability of CC leachate was the highest, and that of WS leachate, which was hardly used by the denitrifying bacteria, was the poorest.

(3) RS, WS, CS, SS, and SH released significant amounts of N, P, and chromatic substances in the initial stage, and RS released the largest amount of secondary pollutants including NH_4_^+^-N [(13.82 ± 0.09) mg/(g·L)], TN [(20.77 ± 1.67) mg/(g·L)], and TP [(4.48 ± 0.25) mg/(g·L)]. The chroma of the WS and CS leachate was higher than that of the others. The N, P, and chromatic substances released from CC were the lowest among the six kinds of agricultural wastes.

(4) The denitrification process could be divided into a start-up phase (1–11 d) and a stable phase (11–27 d). Compared with the other five kinds of agricultural materials, CC had more excellent and stable nitrate and TN removal capacity and better denitrification performance, while less nitrite accumulation and lower effluent NH_4_^+^-N and COD were observed. 

As a result, CC was more appropriate for the solid carbon source for denitrification than the other five kinds of agricultural wastes. In practical use, high nitrate removal efficiency and low secondary pollution risks will be obtained in the nitrate removal of wastewater with a low C/N ratio when CC is applied as the additional carbon source. 

## Figures and Tables

**Table 1:** Contents of C, N, and P of six agricultural wastes.

Materials	C/%	N/%	P/%
RS	69.84	14.38	0.22
WS	72.39	11.02	0.06
CS	62.19	5.34	0.06
CC	51.25	3.22	0.04
SS	43.88	4.59	0.04
SH	61.75	11.00	0.07

**Table 2:** Kinetic fitting results of the carbon release process.

Materials	Second-Order Reaction					Ritger–Peppas		
	Equation	*R* ^2^	*c*_m_	*1/k*	*t*_1/2_	Equation	*R* ^2^	*n*
RS	1/*c* = 0.0006/*t* + 0.0013	0.99	769.23	1666.67	0.46	*M_t_*/*M*_∞_ = 0.7462*t*^0.07^	0.83	0.07
WS	1/*c* = 0.0052/*t* + 0.0045	0.98	222.22	192.31	1.16	*M_t_*/*M*_∞_ = 0.5621*t*^0.13^	0.90	0.13
CS	1/*c* = 0.0015/*t* + 0.0006	0.99	1666.67	666.67	2.50	*M_t_*/*M*_∞_ = 0.5097*t*^0.16^	0.78	0.16
CC	1/*c* = 0.0077/*t* + 0.0029	0.99	344.83	129.87	2.66	*M_t_*/*M*_∞_ = 0.4588*t*^0.18^	0.83	0.18
SS	1/*c* = 0.0098/*t* + 0.0046	0.98	217.39	102.04	2.13	*M_t_*/*M*_∞_ = 0.4479*t*^0.18^	0.91	0.18
SH	1/*c* = 0.0031/*t* + 0.0025	0.99	400.00	322.58	1.24	*M_t_*/*M*_∞_ = 0.5744*t*^0.13^	0.86	0.13

## Data Availability

Data is contained within the article. For detailed information of each part, please contact the corresponding author.

## Supplementary Materials

The following are available online at https://www.mdpi.com/1660-4601/18/3/1232/s1, Figure S1: Release curves of NO3--N from the investigated agricultural wastes.
