# Supplementary figures and images for: Release Mechanism, Secondary Pollutants and Denitrification Performance Comparison of Six Kinds of Agricultural Wastes as Solid Carbon Sources for Nitrate Removal
Source: Int J Environ Res Public Health. 2021 Jan 29;18(3):1232. doi: 10.3390/ijerph18031232 (PMC7908289; doi:10.3390/ijerph18031232)

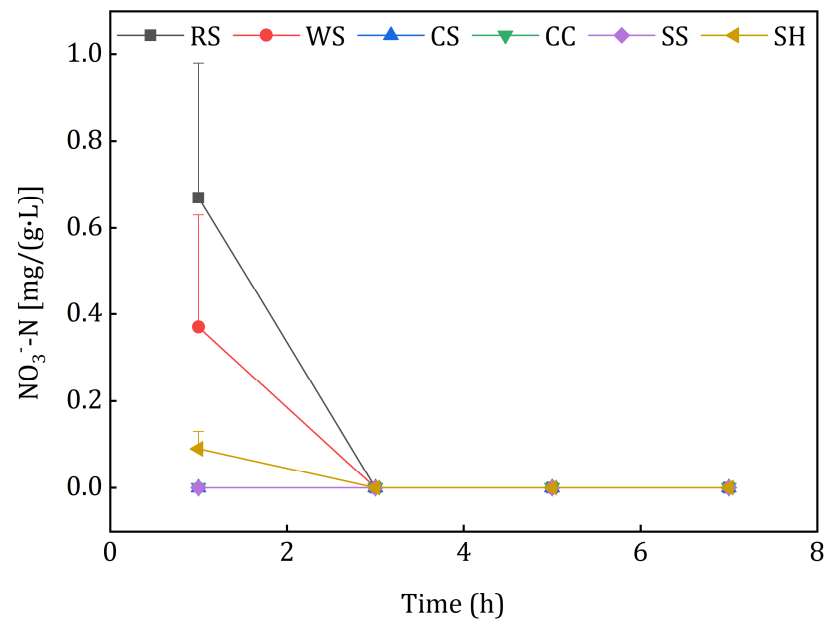

**Figure S1.** Release curves of  $\text{NO}_3^-$ -N from the investigated agricultural wastes

Supplement: Supplementary file 1 [file ijerph-18-01232-s001.pdf]
